# Identification and Characterization of a Rare Fungus, *Quambalaria cyanescens*, Isolated from the Peritoneal Fluid of a Patient after Nocturnal Intermittent Peritoneal Dialysis

**DOI:** 10.1371/journal.pone.0145932

**Published:** 2015-12-30

**Authors:** Chee Sian Kuan, Su Mei Yew, Yue Fen Toh, Chai Ling Chan, Soo Kun Lim, Kok Wei Lee, Shiang Ling Na, Chee-Choong Hoh, Wai-Yan Yee, Kee Peng Ng

**Affiliations:** 1 Department of Medical Microbiology, Faculty of Medicine, University of Malaya, Kuala Lumpur, Malaysia; 2 Department of Medicine, Faculty of Medicine, University of Malaya, Kuala Lumpur, Malaysia; 3 Codon Genomics SB, Selangor Darul Ehsan, Malaysia; Hospital Universitario de La Princesa, SPAIN

## Abstract

Peritonitis is the leading complication of peritoneal dialysis, which is primarily caused by bacteria rather than fungi. Peritonitis is responsible for approximately 18% of the infection-related mortality in peritoneal dialysis patients. In this paper, we report the isolation of a rare fungus, *Quambalaria cyanescens*, from the peritoneal fluid of a man after he switched from continuous ambulatory peritoneal dialysis to nocturnal intermittent peritoneal dialysis. Based on the morphological examination and multigene phylogeny, the clinical isolate was confirmed as *Q*. *cyanescens*. This pathogen exhibited low sensitivity to all tested echinocandins and 5-flucytosine. Interestingly, morphological characterization revealed that *Q*. *cyanescens* UM 1095 produced different pigments at low temperatures (25°C and 30°C) on various culture media. It is important to monitor the emergence of this rare fungus as a potential human pathogen in the tropics. This study provides insight into *Q*. *cyanescens* UM 1095 phenotype profiles using a Biolog phenotypic microarray (PM). Of the 760 nutrient sources tested, *Q*. *cyanescens* UM 1095 utilized 42 compounds, and the fungus can adapt to a broad range of osmotic and acidic environments. To our knowledge, this is the first report of the isolation of *Q*. *cyanescens* from peritoneal fluid, revealing this rare fungus as a potential human pathogen that may be misidentified using conventional methods. The detailed morphological, molecular and phenotypic characterization of *Q*. *cyanescens* UM 1095 provides the basis for future studies on its biology, lifestyle, and potential pathogenicity.

## Introduction

Quambalariaceae is a new family of fungi belonging to the order Microstromatales in the class Exobasidiomycetes [1]. The family comprises the single genus *Quambalaria*. de Beer *et al*. [1] first described the taxonomic changes in plant pathogenic species in the genera *Ramularia* and *Sporothrix* to propose a new genus, *Quambalaria*, to accommodate *Ramularia pitereka* and *Sporothrix eucalypti*. The first *Q*. *cyanescens* isolate (strain CBS 357.73) was recovered from human skin and described as *Sporothrix cyanescens* in 1973 [2]. In 1987, Moore *et al*. [3] showed that the cells of this fungus contain dolipore septa and coenzyme Q-10 and thus reassigned the fungus into a new basidiomycetes genus (*Cerinosterus cyanescens*). Based on a molecular analysis of partial large subunit (LSU)-rDNA, a later study revealed that *C*. *cyanescens* was closely related to *Microstroma juglandis* but differed from other species within the genus *Cerinosterus* [4]. Sigler *et al*. [4] then established the new genus *Fugomyces* and renamed *C*. *cyanescens* as *Fugomyces cyanescens*. The phylogenetic analysis of the internal transcribed spacer (ITS) region in conjunction with the LSU sequences and ultrastructural analysis data reclassified *Fugomyces cyanescens* in the new family Quambalariaceae as *Q*. *cyanescens* [1]. The analysis also confirmed that *Q*. *pitereka*, *Q*. *eucalypti* and *Q*. *cyanescens* are distinct species [1]. To date, the fungal genus *Quambalaria* contains five species, including *Q*. *cyanescens*, *Q*. *pitereka*, *Q eucalypti*, *Q*. *coyrecup* and *Q*. *simpsonii* [1,5–7].

*Q*. *cyanescens* is a hyaline basidiomycete isolated from a broad range of ecological niches, including air, soil, and insect larvae as well as in association with diverse plant sources in various countries [1,2,6,8]. *Q*. *cyanescens* is frequently associated with bark beetles feeding on numerous plants [9]. This fungus, however, is predominantly known as non-pathogenic to plants and has been reported as a symbiont of plants, including *Corymbia* and *Eucalyptus* species [1,6]. Notably, *Q*. *cyanescens* produces the antibiotic sesquiterpene globulol [10]. The crude extract isolated from colored-pigmented endophytic *Quambalaria* species (closely related to *Q*. *cyanescens*) exhibited potent antimicrobial activities against various human and plant pathogens [11,12]. Stodulkova *et al*. [13] described a novel active metabolite naphthoquinone (quambalarine A) with broad antifungal and antibacterial activity from *Q*. *cyanescens*.

*Q*. *cyanescens* is a rare human basidiomycetous pathogen [4,14,15]. The accumulation of data from reported clinical cases suggest that this species is potentially an opportunistic pathogen isolated primarily from immunocompromised or debilitated individuals [4,15]. *Q*. *cyanescens* was also reported as an environmental contaminant associated with pseudoepidemic nosocomial pneumonia [14], and this rare fungus was recently recovered from a female patient after augmentation mammoplasty [16].

We report the first case of *Q*. *cyanescens* isolated from the peritoneal fluid of a patient after nocturnal intermittent peritoneal dialysis. The characteristic morphological features and molecular data identified the fungal strain as *Q*. *cyanescens* UM 1095. The present study provides insight into *Q*. *cyanescens* UM 1095 phenotype profiles, including its nutrient utilization pattern and sensitivity to osmolytes, ions and acidic environments, using the Phenotypic MicroArray (PM) system.

## Materials and Methods

### Ethic statement

Approval for this study was obtained from the Medical Ethics Committee of the University Malaya Medical Centre (reference number: 20157–1519), and written consent was obtained (S1 Fig).

### Clinical history

A 49-year-old male was found to have end-stage renal disease (ESRD) due to hypertensive nephrosclerosis and admitted to the nephrology ward of the Division of Nephrology of UMMC for hemodialysis in April 2014. On 8 December 2014, the patient received continuous ambulatory peritoneal dialysis (CAPD) after Tenckhoff catheter insertion on 21 November 2014. He was recruited to an Automated Peritoneal Dialysis (APD) trial. His APD regime was a two-hour cycle for five cycles per night, using two bags of 5 L 2.5% dialysate. He was stable on CAPD treatment until he changed to nocturnal intermittent peritoneal dialysis (NIPD) treatment using automated cycler machine on 19 December 2014. During NIPD treatment, cloudy peritoneal effluence was observed, but the patient had no symptoms and signs of peritoneal irritation, including fever, abdominal pain, diarrhea or vomiting. He was treated empirically as peritoneal peritonitis and was started on intra-peritoneal (IP) antibiotics, i.e. ceftazidime (IP, 1 g stat and 250 mg 6 hourly) and Cefazolin (IP, 1 g stat and 250 mg 6 hourly). Peritoneal fluid examination showed the presence of 560/μL neutrophils (95% polymorph) and confirmed the diagnosis of peritoneal peritonitis. He demonstrated initial response with reducing peritoneal fluid white cell count from 560/μL to 240/μL. Nonetheless, the peritoneal fluid culture revealed the growth of yeast-like organism and the patient started to receive antifungal therapy (IV fluconazole). He denied any contact with animals and had no recent travel. His Tenckhoff catheter was subsequently removed on 14 January 2015 due to the unresolved peritonitis. He completed 14 days of IV fluconazole and was discharged well. He received CAPD treatment two months after the resolution of peritonitis.

### Fungal isolate

*Q*. *cyanescens* UM 1095 was isolated from the peritoneal dialysis fluid of a dialysis patient with suspected peritonitis after switching from continuous ambulatory peritoneal dialysis to nocturnal intermittent peritoneal dialysis in the Division of Nephrology, University Malaya Medical Centre (UMMC), Kuala Lumpur, Malaysia. The morphological and cultural features of *Q*. *cyanescens* UM 1095 were examined on Sabouraud dextrose agar (SDA), potato dextrose agar (PDA), corn meal agar (CMA) and V8 agar incubated at 25°C, 30°C and 35°C for three days, with alternate-day examination for fungal growth. The macroscopic examination was performed to study the colonial characteristics, including the color of the pigment, texture, and topography. A slide culture was carried out as previously described with slight modifications [17]. The slide cultures were performed by growing the fungus on SDA, PDA, CMA and V8 agar; the slides were examined after 3-day incubation to observe the microscopic structure of the fungus species.

### *In vitro* antifungal susceptibility

The *in vitro* antifungal susceptibility of *Q*. *cyanescens* UM 1095 was examined using the Sensititre YeastOne (TREK Diagnostic System, Cleveland, OH, USA) with a broth microdilution method according to the Clinical and Laboratory Standards Institute (CLSI) M38-A2 guidelines. The isolate was tested against nine antifungal agents (fluconazole, itraconazole, posaconazole, voriconazole, anidulafungin, caspofungin, micafungin, 5-flucytosine and amphotericin B), and the minimum inhibitory concentrations (MICs) were examined after 24-hour incubation.

### DNA sequencing and multilocus phylogenetic analysis

The internal transcribed spacer region (ITS), the small subunit of the ribosomal RNA gene (SSU) and the D1/D2 domain of the 26S rRNA gene were used as targets for the molecular identification of the fungal isolate. Total DNA extraction, PCR amplification, and sequencing were performed as described previously [14,15]. The primers used are listed in S1 Table. The annealing temperatures used to amplify the ITS, SSU and D1/D2 domain of the 26S rDNA genes were 58°C, 52°C and 56°C, respectively. The sequenced data (ITS, SSU, and D1/D2 sequences) were subjected to a BLASTn search against the non-redundant (nr) NCBI-nucleotide database for species identification. Unique ITS and D1/D2 nucleotide sequences from the isolate, together with an additional 14 sequences for the ITS region and D1/D2 domain, respectively, of the *Quambalaria* species were compiled for multilocus phylogenetic analysis (Table 1). *Microstroma juglandis* was used as an outgroup strain in the phylogenetic analysis. Multiple sequence alignments of collected ITS and D1/D2 nucleotide sequences were generated using M-Coffee [18]. Individual alignments were concatenated for Bayesian Markov Chain Monte Carlo (MCMC) analysis partitioned by the gene. Bayesian tree analyses were performed using MrBayes v3.2.2 with reversible jump MCMC averaging over the entire general time reversible (GTR) rates and gamma-distributed rate heterogeneity for all subsets of the partitioned scheme. A total of 1,000,000 generations were run with a sampling frequency of 100, and diagnostics were calculated for every 1,000 generations. The first 2,500 trees were discarded with a burn-in setting of 25%. Convergence was assessed with a standard deviation of split frequencies below 0.01, no noticeable trend for the plot of the generation versus the log probability of the data, and a potential scale reduction factor (PSRF) close to 1.0 for all parameters.

**Table 1:** Details of Isolates Subjected to Multilocus Phylogenetic Analysis.

ITS	D1/D2	Organism name	Strain/Isolate name	Origin	Country	Reference
DQ119134	DQ119136	*Quambalaria cyanescens*	CF 3526	Beetle	Czech	[9]
DQ823421	DQ823441	*Quambalaria cyanescens*	WAC129555	Beetle	Australia	[6]
DQ317623	DQ317616	*Quambalaria cyanescens*	CBS876.73	Beetle	Australia	[1]
DQ823420	DQ823442	*Quambalaria cyanescens*	WAC12954	Beetle	Australia	[6]
DQ823419	DQ823440	*Quambalaria cyanescens*	WAC12952	Plant	Australia	[6]
DQ11913	DQ317615	*Quambalaria cyanescens*	CBS 357.73	Human skin	Netherlands	[1,9]
KF953496	KF953497	*Quambalaria cyanescens*	11PU348	Implant	China	[16]
KT186106	KT186107	*Quambalaria cyanescens*	UM 1095	Peritoneal fluid	Malaysia	This study
DQ823431	DQ823444	*Quambalaria coyrecup*	WAC12947	Plant	Australia	[6]
DQ823430	DQ823448	*Quambalaria coyrecup*	WAC12951	Plant	Australia	[6]
DQ317627	DQ317620	*Quambalaria pitereka*	CMW6707	Plant	Australia	[1]
DQ823423	DQ823438	*Quambalaria pitereka*	DAR 19773	Plant	Australia	[6]
GQ303291	GQ303322	*Quambalaria simpsonii*	CBS:124773	Plant	Thailand	[7]
GQ303290	GQ303321	*Quambalaria simpsonii*	CBS:124772	Plant	Australia	[7]
DQ317625	DQ317618	*Quambalaria eucalypti*	CMW1101	Plant	South Africa	[1]

### Phenotype microarray experiments

The metabolic profile of *Q*. *cyanescens* UM 1095 was evaluated using Biolog PM analysis (Biolog Inc., Hayward, CA, USA). Ten MicroPlate panels (PM1 to PM10), which included carbon, nitrogen, phosphorus, sulfur, nutrient supplements, peptide nitrogen, osmolytes and pH sources, were used in this study. S2 Table summarizes the substrates used in the phenotype microarray experiments.

*Q*. *cyanescens* UM 1095 was grown on SDA at 35°C for 24 hours. The cell suspension was extracted by rolling a sterile, wetted cotton swab over the colonies and resuspended in 15 mL IFY-0 inoculating fluid (Biolog Inc., Hayward, CA, USA). The uniform cell suspension was adjusted to 62% transmission at OD590 nm using a turbidimeter (Biolog Inc., Hayward, CA, USA).

PM1-10 inoculating fluids were prepared according to the manufacturer’s protocol (S3 Table). PM1-10 inoculum suspensions along with different inoculating fluids were prepared as shown in S4 Table. All the wells of PM1-10 were inoculated with 100 μL of the prepared inoculum suspensions and incubated in the Omnilog machine at 35°C for 96 hours. The first well with water was used as a negative control. The experiment was performed in duplicate. The data for each assay from the duplicates were averaged to obtain a mean of the replicates. Based on the 96-hour reading, the heat maps were constructed using Omnilog values ≥150,000.

### Nucleotide sequence accession numbers

The ITS, SSU and D1/D2 domain nucleotide sequences of *Q*. *cyanescens* UM 1095 were deposited in GenBank with accession numbers KT186106, KT186108, and KT186107, respectively.

## Results

### Colonial morphology of *Q*. *cyanescens* UM 1095 on agar

**Fig 1:**
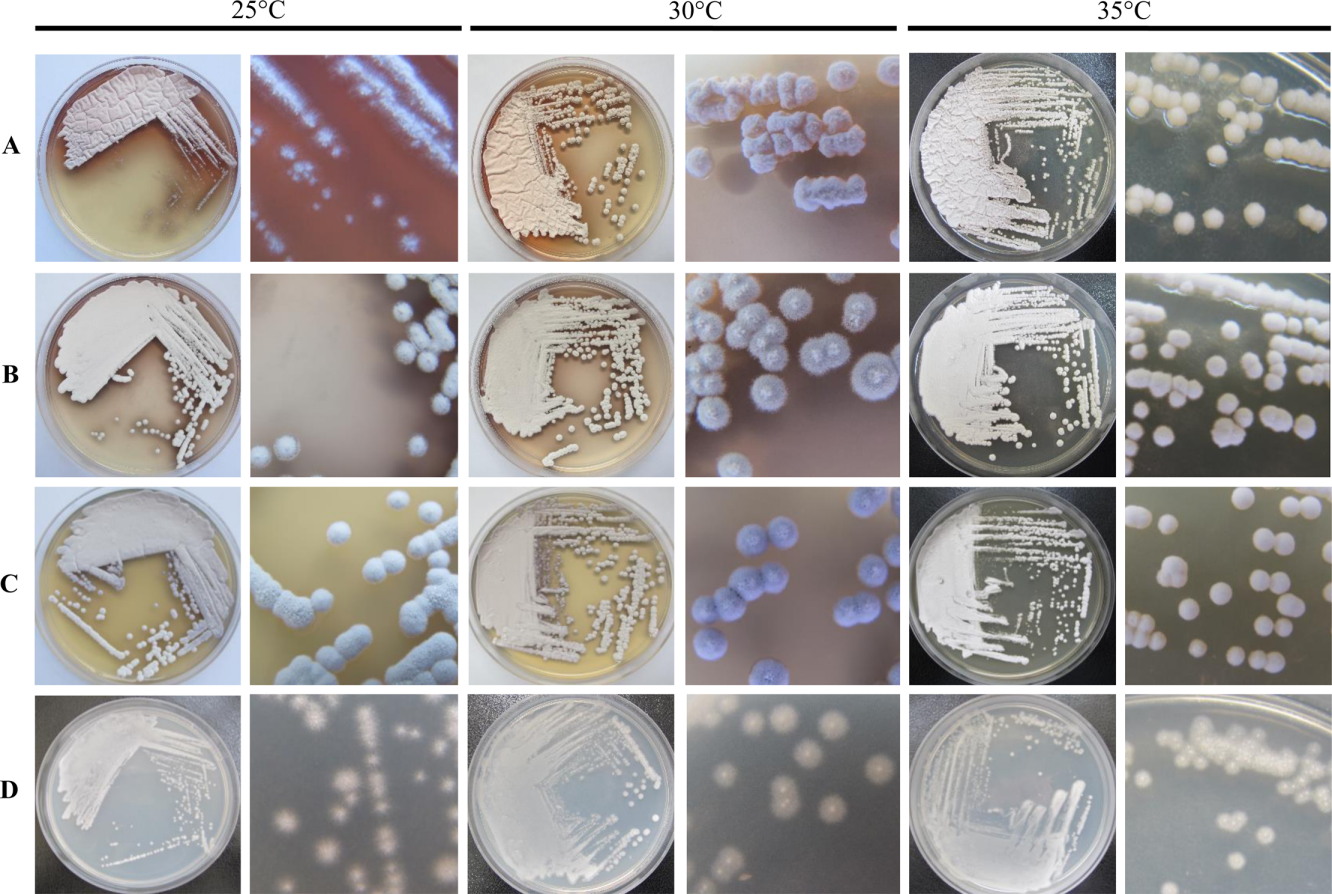
Colonial morphology of *Q*. *cyanescens* UM 1095. The surface and close-up view of the colonial morphology of *Q*. *cyanescens* UM 1095 after being cultured for four days on (A) SDA, (B) PDA, (C) V8 agar and (D) CMA at 25°C, 30°C and 35°C.

The *Q*. *cyanescens* UM 1095 white colonies on SDA were sparse at 25°C but became dense at 30°C and 35°C (Fig 1A). The strain grew more slowly at 25°C and tended to be yeast-like with moist and smooth colonies. At 30°C, the colonies were dried, coarse, and irregular with an umbonate elevation and undulate margin (Fig 1A). At 35°C, the colonies were dried, circular with convex elevation and had an undulate margin. Red pigments were observed diffusing into the agar within 48 hours at 25°C and 30°C on SDA. The production of red pigments was more visible at lower temperatures (25°C and 30°C) than at 35°C (Fig 1A). The diameters of the colonies ranged from 0.05 cm to 0.1 cm, 0.02 cm to 0.3 cm and 0.1 cm to 0.2 cm after 3-day incubation at 25°C, 30°C and 35°C, respectively. Overall, the UM 1095 clinical isolate was found to be morphologically related to *Q*. *cyanescens* based on these characteristics [16].

The *Q*. *cyanescens* UM 1095 isolate grew well on PDA, V8, and CMA at 25°C, 30°C and 35°C. Dark brown and purple pigments were observed diffusing into agar within 48 hours at 25°C and 30°C on PDA and V8, respectively (Fig 1B and 1C). However, no pigment was produced when the fungus was cultured on CMA (Fig 1D). On PDA, yeast-like colonies with butyrous and filiform margins accompanied by a dark brown pigment were observed at 25°C (Fig 1B). At 30°C and 35°C, dense and white circular colonies with an umbonate elevation and filiform margin were observed (Fig 1B). The diameters of colonies ranged from 0.2 cm to 0.3 cm, 0.1 cm to 0.4 cm and 0.05 cm to 0.35 cm after 3-day incubation on PDA at 25°C, 30°C and 35°C, respectively. On V8 agar, yeast-like purple and circular colonies with an umbonate elevation and filiform margin were observed at 25°C and 30°C (Fig 1C). At 35°C, white, circular and creamy colonies with convex elevation and an entire margin were observed (Fig 1C). The diameters of colonies ranged from 0.2 cm to 0.3 cm, 0.05 cm to 0.3 cm, 0.05 cm to 0.18 cm after 3-day incubation at 25°C, 30°C and 35°C, respectively. The *Q*. *cyanescens* UM 1095 colonies on CMA were small, sparse with an umbonate elevation and filiform margin at 25°C, 30°C and 35°C (Fig 1D). The colonial diameters ranged from 0.1 cm to 0.2 cm, 0.003 cm to 0.3 cm, 0.005 cm to 0.2 cm after 3-day incubation on CMA at 25°C, 30°C and 35°C, respectively.

### Microscopic examination of *Q*. *cyanescens* UM 1095

**Fig 2:**
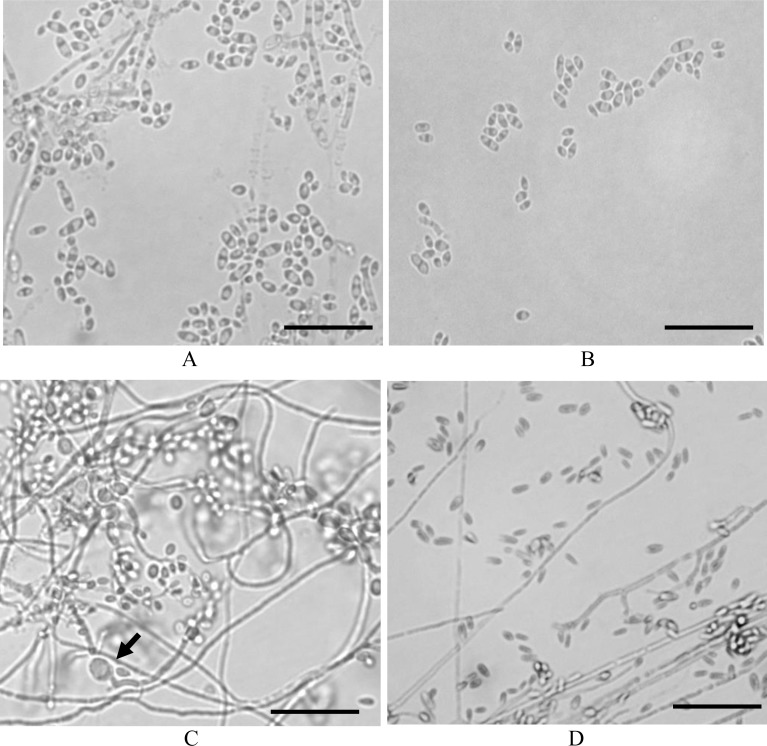
Microscopic morphology of *Q*. *cyanescens* UM 1095. Light micrograph showing the micromorphology of *Q*. *cyanescens* UM 1095 on (A) SDA, (B) PDA, (C) V8 agar and (D) CMA. The chlamydospore is indicated by an arrow (400× magnification, bars 20 μm).

A distinct yeast form of *Q*. *cyanescens* UM 1095 was observed on SDA, PDA, and CMA. Microscopic examination showed a pseudohyphal budding pattern, and the yeast-like cells (1–5 × 1–2 μm) were ovoid, ellipsoid or cylindrical shape with a sympodial conidiogenesis (Fig 2A, 2B and 2D). The filamentous form of *Q*. *cyanescens* UM 1095 was clearly observable on V8 agar, which demonstrated branched and suberected hyphae (Fig 2C). Microscopically, a repeated proliferation of conidiogenous cells was frequently observed. The primary conidia arose by the sympodial growth of the conidiogenous cells and carried several secondary conidia (Fig 2C). In addition, chlamydospores were identified in a culture growing on V8 agar (Fig 2C). No chlamydospores were observed in a culture growing on SDA, CMA, PDA, and V8 agar.

### Sequence-based identification and phylogenetic analysis

The preliminary morphological identification of the *Q*. *cyanescens* UM 1095 isolate was confirmed by PCR amplification of the ITS, D1/D2 domain, and SSU nucleotide sequences, which yielded specific amplification products of approximately 617 bp (ITS), 583 bp (D1/D2 domain) and 1010 bp (SSU), respectively (S2 Fig). By querying ITS and SSU nucleotide sequences against those deposited in the NCBI-nucleotide database, the sequenced ITS of *Q*. *cyanescens* UM 1095 was found to be 100% (617/617 bp) identical to the ITS of *Q*. *cyanescens* strain AUMC 6293 and the SSU was 100% (1010/1010 bp) identical to the SSU of *Q*. *cyanescens* strain CBS 876.73. However, the D1/D2 domain of *Q*. *cyanescens* UM 1095 was 99% (582/583 bp) identical to the D1/D2 domain of *Q*. *simpsonii* culture-collection CBS:124773.

**Fig 3:**
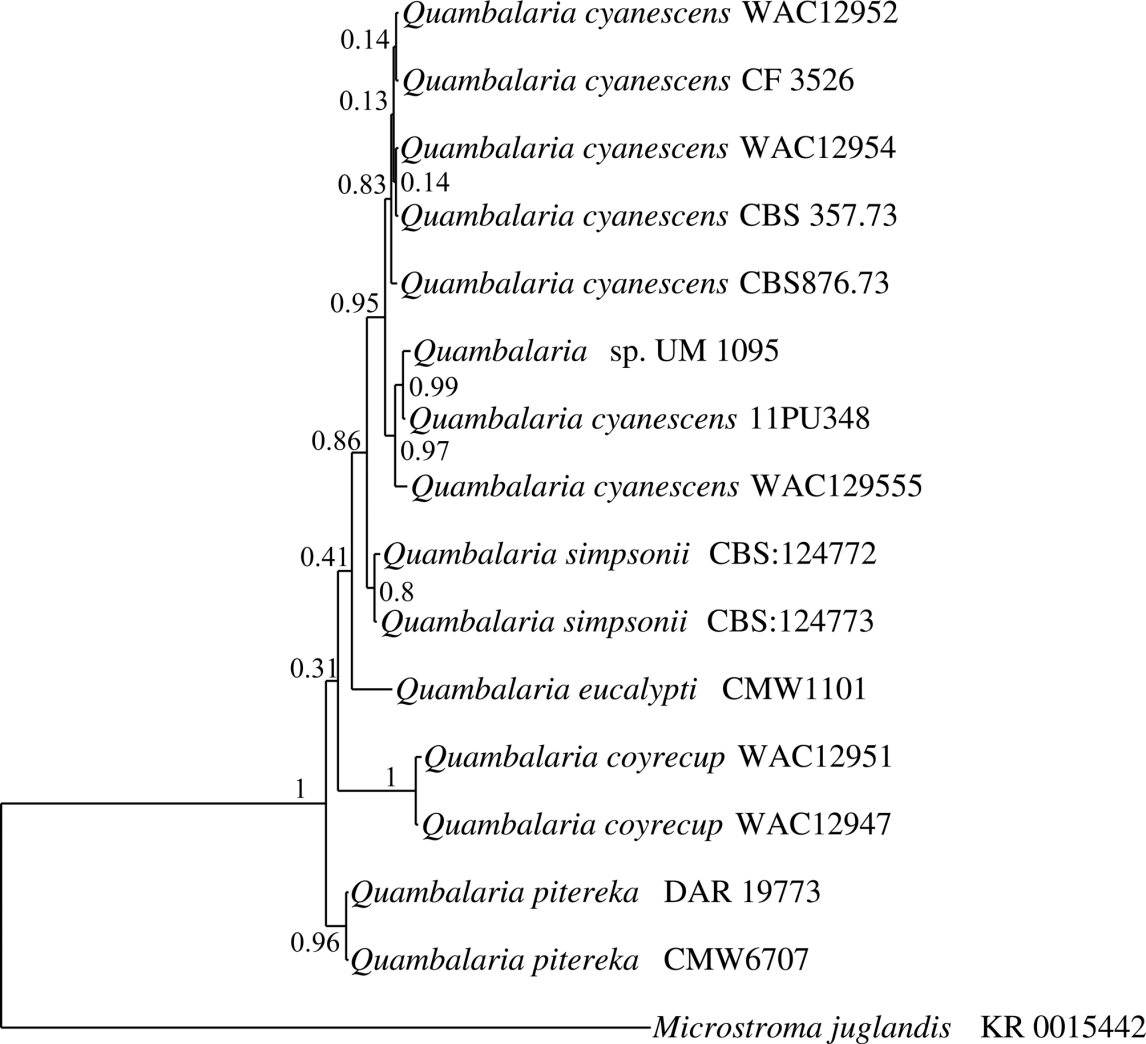
Bayesian phylogram generated using the combined genes of ITS and D1/D2 domain sequence data. The tree was rooted with *M*. *juglandis* as an outgroup. The numbers on the nodes indicate Bayesian posterior probability based on 100 sampling frequencies for a total of 100,000 generations.

The species-level identification of UM 1095 was further confirmed using multilocus phylogenetic analysis. The sequenced ITS and D1/D2 domain gene regions were used to construct a phylogram using combined gene analysis with an additional 14 ex-type strains of the *Quambalaria* species. The SSU region was excluded in the multilocus phylogenetic analysis due to the limited number of *Quambalaria* SSU sequences in the NCBI database. As shown in Fig 3, the five *Quambalaria* species are well separated. Multilocus phylogenetic analysis revealed that UM 1095 was clustered together with *Q*. *cyanescens* strain 11PU348 (Fig 3). Based on the multigene phylogeny, the UM 1095 isolate was confirmed as *Q*. *cyanescens*.

### *In vitro* antifungal susceptibility

*Q*. *cyanescens* UM 1095 is resistant to 5-flucytosine (MIC > 64 μg/mL) and all tested echinocandins, including anidulafungin (MIC > 8 μg/mL), caspofungin (MIC > 8 μg/mL) and micafungin (MIC > 8 μg/mL). *Q*. *cyanescens* UM 1095 is most likely susceptible to amphotericin B (MIC = 1 μg/mL) and fluconazole (MIC = 0.5 μg/mL), itraconazole (MIC = 0.03 μg/mL) and posaconazole (MIC = 0.015 μg/mL).

### Nutrient utilization profile of *Q*. *cyanescens* UM 1095

We performed in-depth investigations of *Q*. *cyanescens* UM 1095 nutritional phenotypic profiles using Biolog PM analysis. Biolog PM metabolic panels (PMs 1–8) contain approximately 200 assays of carbon source metabolism, 400 assays of nitrogen source metabolism, 100 assays of phosphorus and sulfur source metabolism, and 100 assays of biosynthetic pathways (S2 Table). Overall, we observed that nutrient utilization was generally slow over the 4-day incubation.

**Fig 4:**
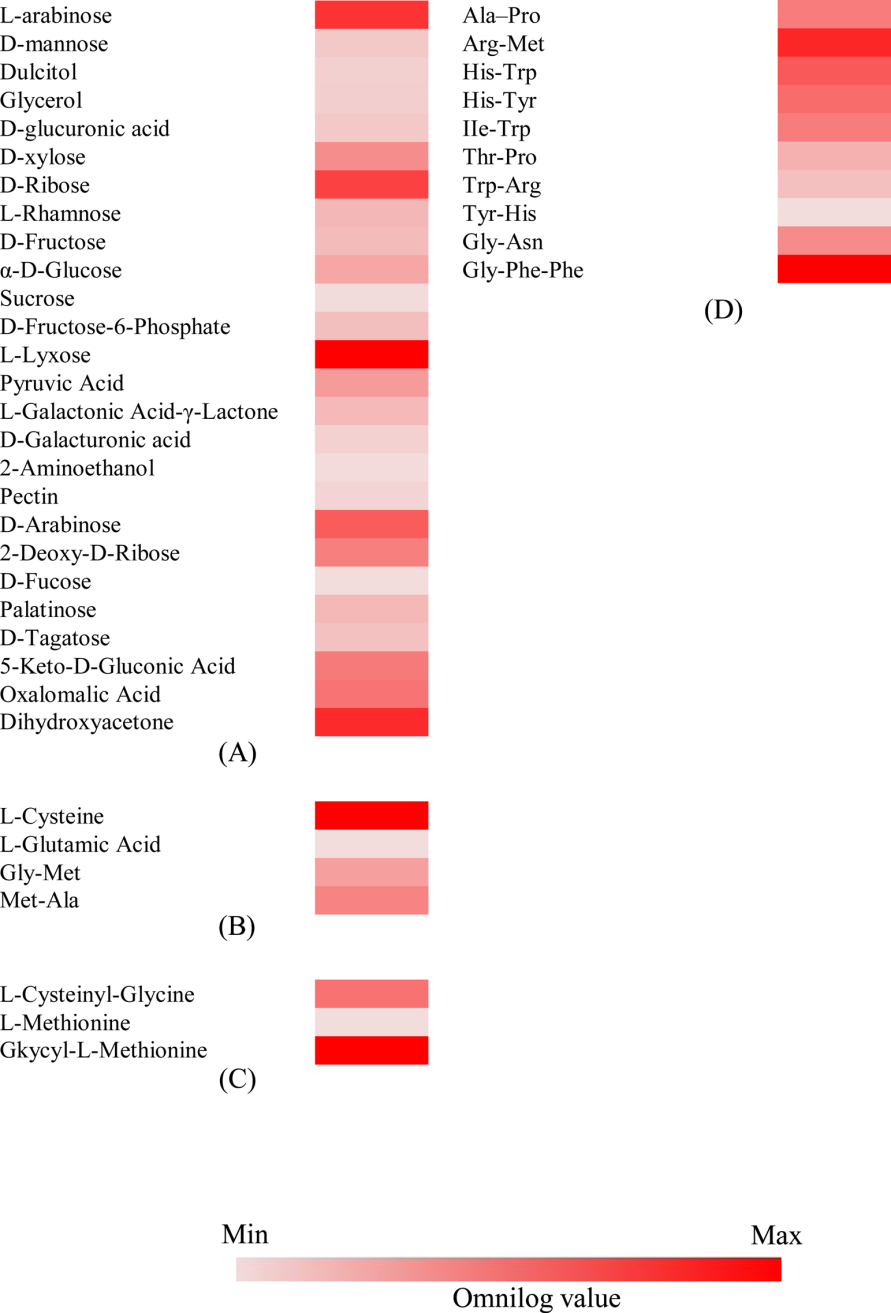
Nutritional profile of *Q*. *cyanescens* UM 1095. (A) Carbon sources. (B) Nitrogen sources. (C) Phosphorus and sulfur sources. (D) Peptide nitrogen sources. Conditions with a final Omnilog unit (at 96 hours) ≥ 15,000 were incorporated into the heat maps. The growth of *Q*. *cyanescens* UM 1095 in a particular substrate over the 96-hour incubation is represented by a color range, as given in the scale bar at the bottom of the figure.

A total of 42 compounds was utilized by *Q*. *cyanescens* UM 1095 of the 760 nutrient sources tested (Fig 4). Of 190 carbon sources (PM1 and PM2) tested, 26 simple sugars and complex carbohydrates were utilized by *Q*. *cyanescens* UM 1095 (Fig 4A). Among the nitrogenous compounds (PM3), L-cysteine (PM3, A11), L-glutamic acid (PM3, A12), glycine-methionine (PM3, H11) and methionine-alanine (PM3, H12) were utilized by *Q*. *cyanescens* UM 1095 (Fig 4B). Furthermore, L-cysteinyl-glycine (PM4, F9), L-methionine (PM4, G7), and glycyl-L-methionine were the only organic phosphorus and sulfur sources utilized by the isolate (Fig 4C). However, no nutritional supplement was utilized by *Q*. *cyanescens* UM 1095. PM analyses revealed that *Q*. *cyanescens* UM 1095 utilized certain peptide nitrogen sources (Fig 4D).

### Chemical sensitivity profile of *Q*. *cyanescens* UM 1095

**Fig 5:**
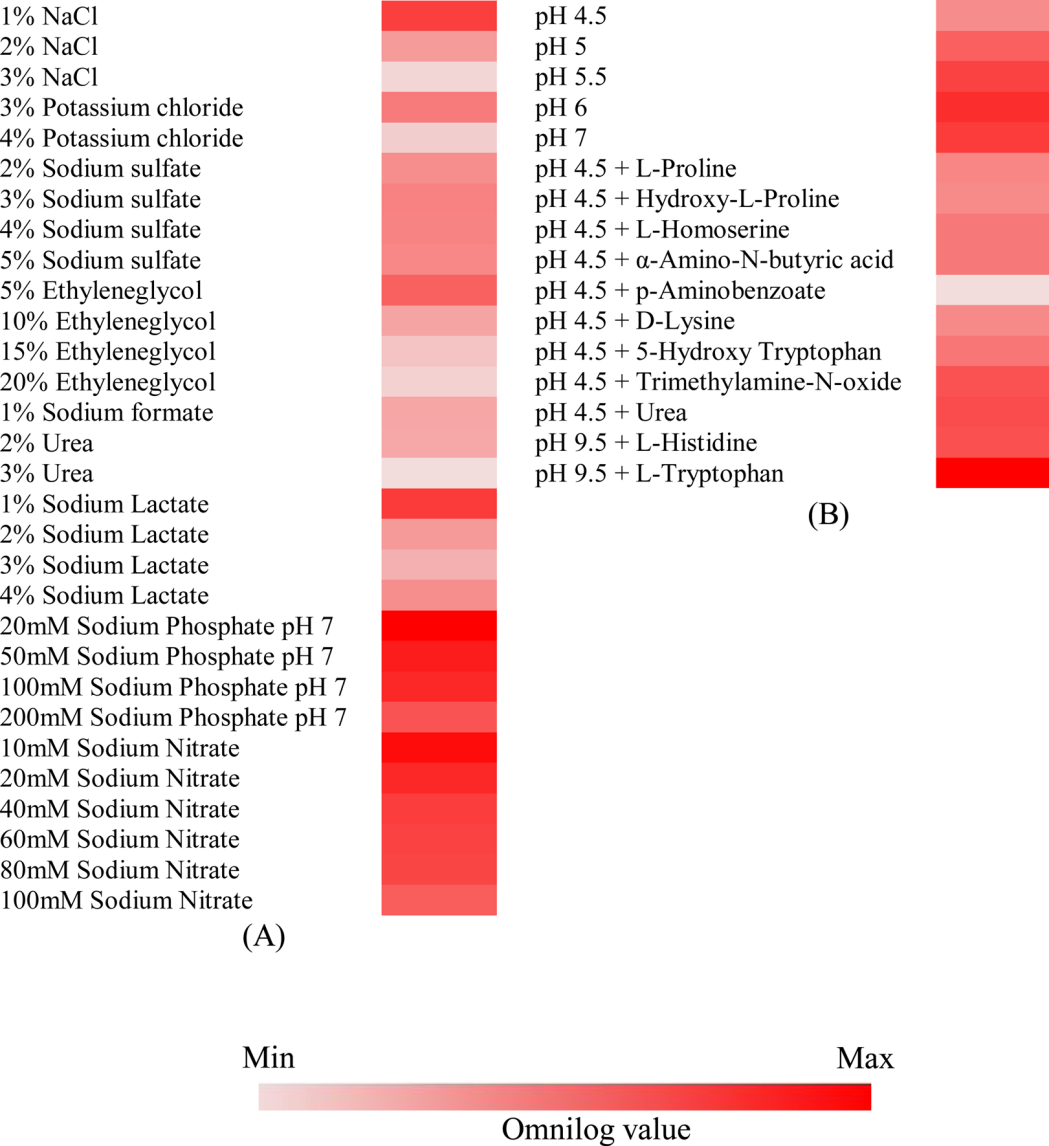
Chemical and pH sensitivity of *Q*. *cyanescens* UM 1095. (A) Osmotic/ions effects. (B) pH effects. Conditions with a final Omnilog unit (at 96 hours) ≥ 15,000 were incorporated into the heat maps. The growth of *Q*. *cyanescens* UM 1095 in a particular substrate over the 96-hour incubation is represented by a color range, as given in the scale bar at the bottom of the figure.

In this study, we found that *Q*. *cyanescens* UM 1095 can adapt to a wide range of osmotic and pH environments. The Biolog PM chemical sensitivity panels (PM9 and PM10) that were used in this study consisted of approximately 100 assays of ion effects and osmolarity, 100 assays of pH effects and pH control with deaminases and decarboxylases (S2 Table). Each chemical sensitivity assay included at least four increasing doses of the test chemical. For sensitivity to osmolytes, *Q*. *cyanescens* UM 1095 was found to be very sensitive to several sodium salts with detectable growth, including sodium formate (2–6%), sodium benzoate pH 5.2 (20–100 mM) and sodium nitrite (10–100 mM; Fig 5A). In addition, PM analysis revealed that 10 mM ammonium sulfate was toxic for *Q*. *cyanescens* UM 1095 (Fig 5A). However, *Q*. *cyanescens* UM 1095 was capable of growing in potassium salt concentrations of 3–6%. The fungus was also found to be able to grow in other osmolytes and ions such as sodium sulfate, ethylene glycol, sodium phosphate and sodium nitrate (Fig 5A). Moreover, the results showed that *Q*. *cyanescens* UM 1095 can grow in an acidic environment, with maximum growth between pH 4.5 and 7 (Fig 5B).

## Discussion

Peritonitis is the main complication of peritoneal dialysis and is mostly caused by bacterial pathogens. An estimated 18% of the infection-related mortality in peritoneal dialysis patients is attributed to peritonitis [19]. By comparison, fungi only account for 1% to 15% of peritonitis [11,20–22]. However, fungal peritoneal dialysis-associated infections are commonly caused by *Candida* species and are usually associated with significant morbidity and mortality [23,24]. Various molds and yeasts, such as *Aspergillus* sp., *Penicillium* sp., *Paecilomyces* sp., *Trichosporon* sp., *Rhodotorula* sp. and *Cryptococcal* sp., are occasionally reported [22,25–27]. Nevertheless, polymicrobial peritonitis that is caused by either fungi or bacteria is a predictor of a poor response to antibiotic therapy [12]. Peritoneal dialysis-associated infection is a common cause of patients discontinuing peritoneal dialysis and switching to hemodialysis [19]. Hamai *et al*. [28] reported that there is a risk of peritonitis introduced by contamination from domestic pets. The case study showed that a nocturnal intermittent peritoneal dialysis patient was infected with unusual bacterial pathogens, including *Enterobacter agglomerans*, *Pasteurella multocida* and *alpha-streptococcus*, most likely due to transmission from the patient’s domestic cats [28]. In the present study, we isolated a rare clinical basidiomycetous fungus, *Q*. *cyanescens*, from the peritoneal fluid of a patient who switched from continuous ambulatory peritoneal dialysis to nocturnal intermittent peritoneal dialysis. This fungus has been isolated from heart transplant patients [15], lymphoma patients [4] and augmentation mammoplasty patients [16]. However, no apparent clinical evidence of infection was shown in these studies. Jackson *et al*. [14] proposed that *Q*. *cyanescens* may be an environmental contaminant. In the present study, the *Q*. *cyanescens* UM 1095 may be as an emerging pathogen to cause real infection or foreign-body reaction but not an environmental contaminant because i.) the patient denied to be contacted with any animals ii.) no other bacteria or fungi were isolated from the Tenckhoff catheter tip, iii.) the white blood cell count in the cloudy peritoneal effluent of the patient was abnormally high, and iv.) no other organism was isolated from the peritoneal fluid. However, at this stage of knowledge, the pathogenic role of *Q*. *cyanescens* in peritonitis remains unknown, although it is a low-virulence human pathogen [4].

At the initial stage, we failed to identify *Q*. *cyanescens* UM 1095 based on basic microbiological examination. Gram staining showed no bacteria but only fungal elements. On *Brilliance Candida* agar, we obtained dark blue and yeast-like colonial morphology and identified the isolate as a *Candida* species, as in a previous study [16]. The API 20C Aux yeast identification system (bioMérieux, Hazelwood, MO, USA) misidentified the isolate as *Rhodotorula mucilaginosa*, which did not correspond to the culture characteristics. This failure of accurate identification of our isolate identity was most likely due to the absence of a *Quambalaria* species database in the identification system. When standard assimilation tests fail to determine the isolate identity, species-level identification was achieved through specific culture characteristics and molecular characterization. Subsequent colonial morphological examination and multilocus phylogenetic analysis identified the clinical isolate as *Q*. *cyanescens*. In contrast to a previous study [16], multilocus phylogeny using the combined ITS and D1/D2 sequences not only was able to distinguish the distinct five species within the *Quambalaria* genus but also clustered our isolate with *Q*. *cyanescens*.

The main aim of peritonitis treatment is to resolve inflammation by eradicating the causative pathogen(s). The early and rapid identification of causative pathogen(s) is important with the advent of clinical management to ensure good clinical outcomes and to reduce the unnecessary overuse of antimicrobial agents. In this study, we found that *Q*. *cyanescens* UM 1095 had the same resistance profile as *Q*. *cyanescens* 11PU348, which exhibited high MICs to three compounds of the echinocandin class of drugs and 5-flucytosine. Echinocandin resistance is considered rare, although a few studies have shown echinocandin resistance in *Candida albicans*, *C*. *parapsilosis*, *C*. *tropicalis*, *C*. *guilliermondii* and *C*. *kefyr* [29–31]. In the literature, echinocandin resistance in *Candida* species is associated with the mutations in two hot spot regions of *FKS* genes, which encode the major subunit of the 1,3-D-glucan synthase complex [32–35]. However, the molecular basis underlying echinocandin resistance in *Q*. *cyanescens* UM 1095 is unknown.

All microbial pathogens require a variety of nutrient sources, including carbon, nitrogen, phosphorus and sulfur, for growth and proliferation. Here, we performed comprehensive phenotypic characterization and metabolic profiling of this rare clinical isolate using Biolog PM to provide a better understanding of its nutritional requirements, which are likely needed for it to grow. A closer analysis of the Biolog data revealed that *Q*. *cyanescens* UM 1095 has a high capacity to utilize a number of simple sugars and complex carbohydrates such as pectin and glycerol. This high variability may explain why *Q*. *cyanescens* is the only species of the *Quambalaria* genus that is found in virtually all habitats, including plants, insects, and humans. However, we observed that *Q*. *cyanescens* UM 1095 utilizes a narrow spectrum of nitrogen, phosphorus, sulfur and peptide nitrogen sources. The most striking finding was the high metabolic versatility of *Q*. *cyanescens* UM 1095 to grow and adapt to a broad range of osmotic environments. This adaptability may be an important contributing factor to its pervasive ability to grow in diverse unfavorable environments. As previously noted [36], 3% NaCl is the optimal condition for the growth of *Hortaea werneckii*, a causative agent of tinea nigra. However, Kogej *et al*. [37] showed that 3% NaCl is a concentration that is toxic to *Saccharomyces cerevisiae*. In the present study, PM analysis showed that *Q*. *cyanescens* UM 1095 is capable of growing well in NaCl concentrations of 1–3%. The ability of *Q*. *cyanescens* UM 1095 to utilize various osmolytes showed that the fungus has a good osmoadaptation system to tolerate osmotic stress, which merits further study.

## Conclusion

In this study, we isolated a rare clinical fungal pathogen: *Q*. *cyanescens* UM 1095. This is the first reported isolation of *Q*. *cyanescens* from peritoneal fluid. Based on the macroscopic examination on agar, we observed that the clinical isolate exhibits a mycelium-yeast dimorphism characteristic, which is typical of *Q*. *cyanescens*. Multilocus phylogenetic analysis revealed that *Q*. *cyanescens* UM 1095 is tightly clustered with the *Q*. *cyanescens* strain 11PU348, which was also isolated from humans. In this study, Biolog PM was used to characterize the phenotypic profile of *Q*. *cyanescens* UM 1095. Comprehensive phenotypic characterization and metabolic profiling of this rare species enable the design of interventions to prevent the proliferation of this potential pathogen. The Biolog PM may be a effective diagnostic tool, especially when combined with multilocus phylogenetic analysis. We hope that the in-depth analysis of the *Q*. *cyanescens* UM 1095 phenotypic profile will help to decipher its basic biology and provide a valuable platform for future fungal research.

## Supporting Information

S1 Fig Witten consent obtained from the patient in the study. (PDF)

S2 Fig PCR amplification of *Q*. *cyanescens* UM 1095 ITS, D1/D2 domain and SSU nucleotide sequences. M1: 100 bp ladder (i-DNA Biotechnology); M2: Lambda DNA/HindIII Marker (Fermentas).
(TIF)

S1 Table Primers used for PCR amplification. (XLSX)

S2 Table The full list of substrates assayed in the PM experiments. (XLSX)

S3 Table Final concentrations of ingredients in PM inoculating fluids. (XLSX)

S4 Table Preparation of PM1-10 inoculum suspensions. (XLSX)
