# Supplementary material for: Identification and Characterization of a Rare Fungus, Quambalaria cyanescens, Isolated from the Peritoneal Fluid of a Patient after Nocturnal Intermittent Peritoneal Dialysis
Source: PLoS One. 2015 Dec 30;10(12):e0145932. doi: 10.1371/journal.pone.0145932 (PMC4696669; doi:10.1371/journal.pone.0145932)
Supplement: S1 Fig — (PDF) [file pone.0145932.s001.pdf]

CONSENT BY PATIENT FOR OPERATION/ TREATMENT/PROCEDURE

UNIVERSITY MALAYA MEDICAL CENTRE

I ______________________, Identification Number ______________________
(Name of Patient)

of ____
(Address)

hereby consent to the * ~~operation/treatment/procedure of~~ Case report to Journal

the nature, purpose, effects and risks which have been explained to me by Dr. Lim soo Kun
(Name of Attending Doctor)

through the interpretation of — in Mandarin
(Name of Interpreter)
language /dialect.

The doctor has explained the following (Tick whichever applies)

- [ ] The nature of the operation/treatment/procedure.
- [ ] Sedation need to be given.
- [ ] The reason for the operation/treatment/procedure.
- [ ] The level of expertise used during the operation/treatment/procedure.
- [ ] The benefits of the operation/treatment/procedure.
- [ ] The general and specific risks of the operation/treatment/procedure.
- [ ] The alternatives to the operation/treatment/procedure.
- [ ] The line of action in the event of a complication arising following the operation/treatment/procedure.
- [ ] The patient's freedom of choice.
- [ ] The patient's freedom to ask questions.
- [ ] The patient's freedom to withdraw consent at any time before the procedure is performed.

Additional information discussed between doctor and patient (or as attached).

Patient was informed and explained that his peritonitis case will be reported to a peer-reviewed journal. Patient understood & was agreeable

Signature : [signature] (Patient) Date : 26-12-2015

Signature : [signature] (Attending Doctor) Stamp: A/PROF DR LIM SOO KUN
MMC Full Reg. no. 36871
Consultant Nephrologist & Physic[illegible]
Department of Medicine
UMMC

Date : ____________

Signature: [signature] (Witness)
Rohana Hisham (Name) Date: 26/12/2015

* **Strike out where not applicable**

32210880 L 28/02/1978
WONG TECK SENG
780228145231 Wad/Klk : 8TE
32210880

CONSENT BY PATIENT FOR OPERATION/ TREATMENT/PROCEDURE

BK-MIS-118-E02
