# Supplementary figures and images for: Identification and Characterization of a Rare Fungus, Quambalaria cyanescens, Isolated from the Peritoneal Fluid of a Patient after Nocturnal Intermittent Peritoneal Dialysis
Source: PLoS One. 2015 Dec 30;10(12):e0145932. doi: 10.1371/journal.pone.0145932 (PMC4696669; doi:10.1371/journal.pone.0145932)

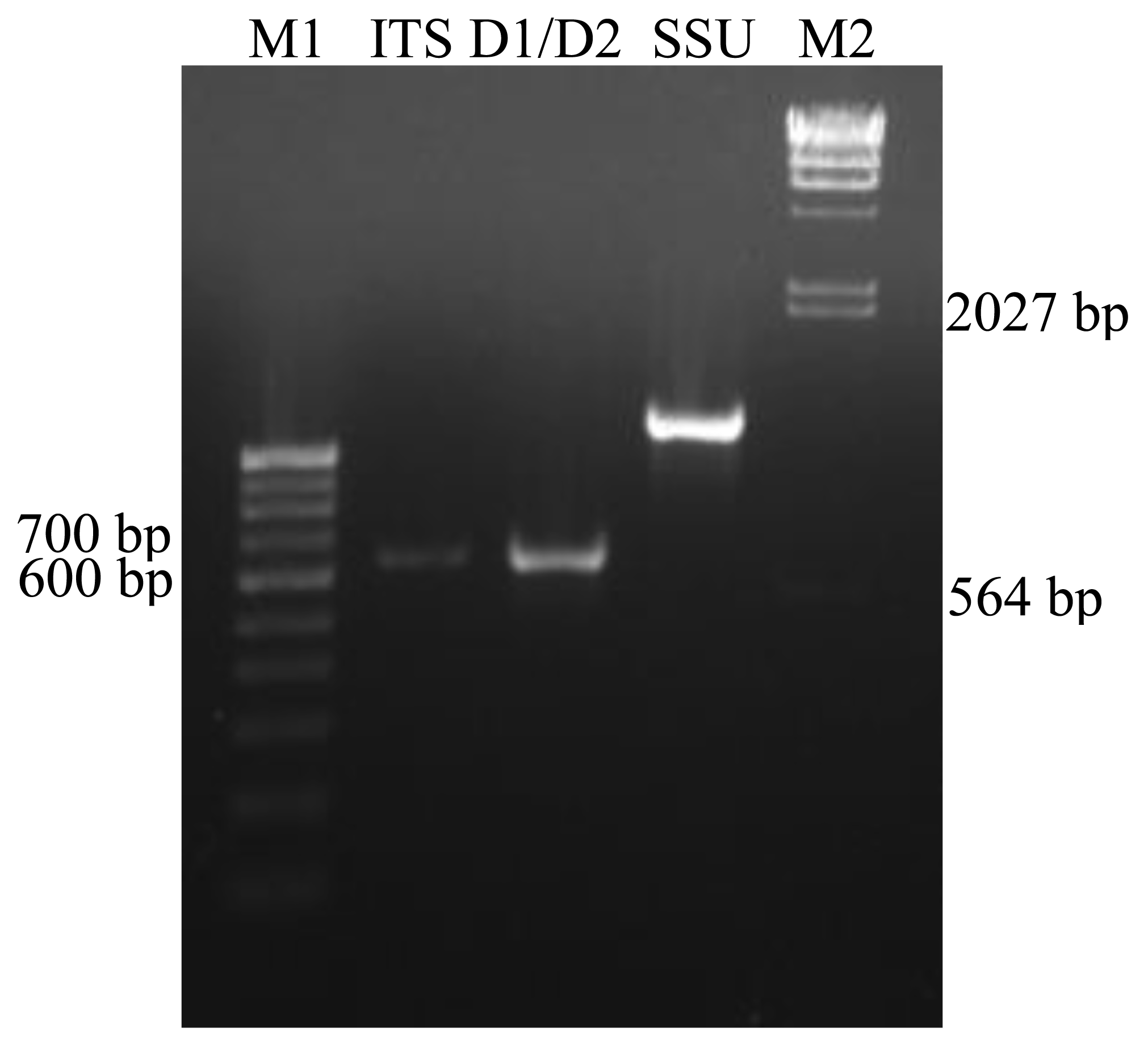

Supplement: S2 Fig — M1: 100 bp ladder (i-DNA Biotechnology); M2: Lambda DNA/HindIII Marker (Fermentas). (TIF) [file pone.0145932.s002.tif]
